# PROXIMAL METASTATIC GASTRIC CANCER IN YOUTH: THE MAYOR OF SÃO PAULO CITY AS AN EXAMPLE OF A CURRENT PHENOMENON

**DOI:** 10.1590/0102-672020200003e1536

**Published:** 2020-12-16

**Authors:** Marcus Fernando Kodama Pertille RAMOS, Leandro Cardoso BARCHI, Antonio Carlos WESTON, Bruno ZILBERSTEIN

**Affiliations:** 1 Brazilian Gastric Cancer Association, São Paulo, SP, Brazil; 2 Cancer Institute, Hospital das Clínicas, School of Medicine, University of São Paulo, São Paulo, SP, Brazil; 3 Santa Casa de Misericórdia of Porto Alegre, Porto Alegre, RS, Brazil

The recent news of the diagnosis of gastric cancer (GC) affecting the mayor of the city of São Paulo brought this disease again to the focus of the news. In addition to the personal drama this diagnosis causes in all patients, some features of this particular case have gained much prominence in the media. Widespread information reported that it was a GC in a patient under 40 years of age, proximally located, with liver metastasis who will initially be treated with chemotherapy. These features have recently been studied in national publications that deserve review by readers^4^ ^,^ ^5^.

The mean age of patients diagnosed with gastric cancer is 65 years and about 5% are younger than 45 years^3^ ^,^ ^4^. The trend of falling overall incidence of GC is not so observed in young patients which lead to an increase in the proportional incidence of tumors in young people. Tumors in young adults tend to be diffuse, with aggressive behavior, but age alone is not considered a poor prognostic factor. Tumors in young people always raise the possibility of hereditary component; however, less than 3% of gastric tumors are associated with hereditary genetic syndromes. These syndromes include hereditary diffuse gastric cancer (HDGC), Li-Fraumeni, familial adenomatosis polyposis, Lynch and Peutz-Jeghers. Clinical criteria for suspected HDGC diagnosis include: 1) families with two or more GC patients of any age being at least one diffuse type; 2) individuals with diffuse GC under 40 years of age; 3) families diagnosed with at least one case of diffuse GC or breast lobular tumor before age 50. It is noteworthy that the presence of these criteria does not confirm the diagnosis of HDGC, only suggests the search for mutation in these cases. 

Proximal tumor localization reflects another recent trend that is already well established worldwide^1^ ^,^ ^3^. The carcinogenesis of distal gastric lesions involves chronic inflammation mainly associated with alcohol consumption, smoking and *H.pylori* infection^2^. These factors also have an effect on proximal lesions; however, the increase in the incidence of obesity in the population seems to be fundamental for the increase in incidence of this type of tumor. While gastric tumors are becoming more proximal, esophageal tumors are becoming more distal. Squamous cell carcinoma in the middle esophagus is becoming less frequent compared to distal esophageal adenocarcinomas. Thus, the esophagogastric transition is the region where we expect to find more and more lesions.

The presence of metastasis in the diagnosis of any tumor is always a factor of poor prognosis. However, the recent evolution of chemotherapy treatments has allowed the possibility of adopting conversion therapy in selected cases. Conversion therapy is defined as the use of chemotherapy for metastatic or unresectable tumors in the initial diagnosis. After initial chemotherapy treatment, patients with a good response where R0 resection is feasible are referred for surgery. About 30% of patients who initiate this approach are effectively operated after initial chemotherapy. In cases with resected tumors R0, increased survival and even reports of cure are observed.

 These reports led our service to publish initial experience of 16 cases^4^. Updating our series, we have performed three more resections after conversion therapy and we have three patients with disease-free survival over 36 months. These are certainly rare cases, but they exist and inspire us to always seek the best options for patients. 

In this context of difficulty and struggle for cure, it is only fair to remember the motto of the city of São Paulo: *NON DUCOR, DUCO* (I’m not lead, I lead). 
